# A Method to Prioritize Quantitative Traits and Individuals for Sequencing in Family-Based Studies

**DOI:** 10.1371/journal.pone.0062545

**Published:** 2013-04-23

**Authors:** Kaanan P. Shah, Julie A. Douglas

**Affiliations:** 1 Department of Human Genetics, University of Michigan Medical School, Ann Arbor, Michigan, United States of America; National Taiwan University, Taiwan

## Abstract

Owing to recent advances in DNA sequencing, it is now technically feasible to evaluate the contribution of rare variation to complex traits and diseases. However, it is still cost prohibitive to sequence the whole genome (or exome) of all individuals in each study. For quantitative traits, one strategy to reduce cost is to sequence individuals in the tails of the trait distribution. However, the next challenge becomes how to prioritize traits and individuals for sequencing since individuals are often characterized for dozens of medically relevant traits. In this article, we describe a new method, the Rare Variant Kinship Test (RVKT), which leverages relationship information in family-based studies to identify quantitative traits that are likely influenced by rare variants. Conditional on nuclear families and extended pedigrees, we evaluate the power of the RVKT via simulation. Not unexpectedly, the power of our method depends strongly on effect size, and to a lesser extent, on the frequency of the rare variant and the number and type of relationships in the sample. As an illustration, we also apply our method to data from two genetic studies in the Old Order Amish, a founder population with extensive genealogical records. Remarkably, we implicate the presence of a rare variant that lowers fasting triglyceride levels in the Heredity and Phenotype Intervention (HAPI) Heart study (p = 0.044), consistent with the presence of a previously identified null mutation in the *APOC3* gene that lowers fasting triglyceride levels in HAPI Heart study participants.

## Introduction

The genetic architecture of most complex traits and diseases is poorly understood. Indeed, genome-wide association studies (GWAS’s) have identified hundreds of loci with relatively weak effects on complex traits and diseases, leaving much of their heritability unaccounted for [1]. This is expected (in part) since the genotyping technology used in these studies captures primarily common sequence variation, namely, single nucleotide polymorphisms (SNPs) with minor allele frequencies (MAFs) of at least 5%. Rare variants (MAF<5%), which are poorly captured by standard GWA arrays [2], may have larger effect sizes than common variants and may make an important contribution to complex traits and diseases. In fact, results from large-scale sequencing studies (n>10,000) suggest a much higher load of rare variants than was previously appreciated and may bear on the heritability unexplained by GWAS [3], [4].

Recent advances in DNA sequencing technology have dramatically increased the capacity to discover rare variants. However, it is still cost prohibitive to sequence whole genomes (or even whole exomes) on the scale of a GWAS, e.g., by sequencing all study participants. For studies of quantitative traits, one strategy to reduce cost is to sequence individuals with extreme phenotypes. Simulation studies [5], [6] and empirical studies of candidate genes suggest that this is a powerful approach for identifying rare trait-associated alleles. For example, this approach has been successfully used to identify rare variants in candidate genes associated with body mass index (BMI) [7], high-density lipoprotein (HDL) [8], low-density lipoprotein (LDL) [9], [10], and sterol absorption [9].

The power of extreme-trait sequencing or selective genotyping, originally introduced by Lander and Botstein [11], derives from the fact that rare trait-influencing alleles with modest to large effects will be enriched in frequency in the upper or lower tail of the trait distribution. The success of this strategy, however, depends (in part) on the careful selection of traits and individuals to sequence. In theory, the most powerful approach is to select and sequence the most extreme individuals from each tail of the trait distribution. In practice, however, power may be lost by sequencing too few or too many individuals or by choosing a suboptimal trait. To optimize the selection of traits and individuals for an extreme-trait sequencing study, we develop a new statistical test, the Rare Variant Kinship Test (RVKT). Our test is designed for use in family-based studies in which individuals have already been phenotyped – but not necessarily genotyped – for dozens of quantitative traits relevant to human health and disease.

Briefly, the RVKT leverages the relatedness of individuals in family-based studies to identify quantitative traits that are most likely to be influenced by rare variants. The premise of our test is that rare variants with at least modest effects will be enriched in the tails of the trait distribution and preferentially carried by closely related individuals. Unlike complex segregation analysis, which attempts to identify a particular mode of inheritance, our approach makes few assumptions about the trait architecture. We assess the power of our test via simulation and apply it to dozens of quantitative traits from two of our studies in the Old Order Amish population.

## Methods

### Ethics Statement

All human subject research was previously reviewed and approved by the Institutional Review Boards at the University of Michigan and the University of Maryland. Written informed consent was obtained from all study participants.

### Overview

Here we describe the RVKT, simulations to assess power, and applications to two family-based studies. The RVKT requires a sample of families with pedigree and phenotype data and assumes that each of the quantitative traits to be tested has a narrow-sense heritability that is significantly different from zero. The null hypothesis of the RVKT is that a given trait is purely polygenic, meaning influenced by multiple additive, independent loci of small effect. Under the null hypothesis, individuals in the tail of the trait distribution carry trait-influencing alleles at many loci. The alternative hypothesis of the RVKT is that at least one locus of modest to large effect influences the trait, and accordingly, that the trait-associated allele(s) is necessarily rare (the rare variant). Under the alternative hypothesis, individuals in the tail of the trait distribution should preferentially carry the rare variant and thus may be more closely related when measured against the null hypothesis.

### The Rare Variant Kinship Test

For each trait, we define and calculate the RVKT statistic as the mean of the pair-wise kinship coefficients between individuals in the tail of the quantitative trait distribution. Tail membership is determined by ordering individual trait values. Conditional on the pedigrees in the sample, the kinship coefficient between two individuals is the probability that a randomly chosen allele from one individual and a randomly chosen allele from the other individual at an autosomal locus are inherited identical by descent from a recent, common ancestor. We calculate pair-wise kinship coefficients using the matrix method described by Lange [12] and implemented in MENDEL version 10.0.0. Since the kinship coefficient depends only on the structure of the pedigree connecting a pair of individuals, the RVKT requires pedigree data but no genetic data. Thus, it can be applied before carrying out expensive genotyping or sequencing experiments.

To assess statistical significance, we compare the observed RVKT statistic for each trait to its expected distribution under a purely polygenic model (the null hypothesis) (described below). Under the alternative hypothesis, the observed RVKT statistic may exceed its expected value, meaning individuals in the tail of the trait distribution may be more closely related than expected under the null hypothesis. Thus, we use a one-sided test. Because the genetic architecture of each trait is unknown, we conduct the RVKT for both tails of the trait distribution (upper and lower) and multiple tail sizes. Tail size is the proportion of individuals in the tail of the trait distribution. We then select the RVKT statistic with the minimum p-value in each tail (p_min_).

The expected distribution of the RVKT statistic depends on the actual pedigrees and the narrow-sense heritability of the trait. Thus, we use simulation to generate an empirical null distribution for each trait. Specifically, using MORGAN version 3.0 [http://www.stat.washington.edu/thompson/Genepi/MORGAN/Morgan.shtml], we simulate 10,000 replicates of a purely polygenic trait with heritability equal to the narrow-sense heritability estimated from the observed data. Simulations are done conditional on the observed pedigrees. We calculate the RVKT statistic for each replicate using the same tail sizes tested in the observed data. The resulting RVKT statistics form an empirical null distribution for each trait and tail size. From this distribution, we determine a rejection region based on the prescribed size of the test (false-positive rate).

### Assessment of the Test by Computer Simulation

To evaluate the power of our test, we conducted gene dropping simulations conditional on our Amish pedigrees (described below), and for comparison, four-person nuclear families (two parents and two offspring) with sample sizes corresponding to our Amish studies. Specifically, we simulated a single additive, bi-allelic locus with a trait-influencing allele frequency of 0.5, 1, 2, 3, or 4% (the rare variant) that accounted for 2, 5, 10, 20, or 30% of the total trait variance. In each simulation, we assumed that multiple additive, independent genetic factors, including the rare variant, accounted for 40, 60, or 80% of the trait variance (the narrow-sense heritability). For each set of parameters, we simulated 1,000 replicates using MORGAN version 3.0 and tested tail sizes of 1, 2, 4, 6, and 8%. For each tail size, power was calculated as the proportion of replicates for which the RVKT statistic equaled or exceeded the 95^th^ percentile of the empirical null distribution, i.e., using a significance level of 0.05. We generated a single null distribution (as described above) for narrow-sense heritabilities of 40, 60, and 80% and repeatedly compared each replicate under the alternative hypothesis to this distribution.

A subset of the simulations above were conducted on pedigree structures connecting 1,481 women from our genetic study of mammographic density [13] and 868 men and women from the Heritability and Phenotype Intervention (HAPI) Heart study, a genetic and environmental study of cardiovascular risk factors [14]. Individuals in both studies were from the Old Order Amish population of Lancaster County, Pennsylvania. Using the extensive genealogical information available from the Anabaptist Genealogical Database [15], [16], we were able to connect subjects within each study into a single, 13-generation pedigree. Table 1 gives the number and types of pair-wise relationships after merging in only two generations from the complete pedigree, i.e., by merging in the parents and grandparents of all study subjects, and trimming the resulting pedigrees using PedCut [17] with a maximum bit size of 100. To assess the impact of pedigree complexity on power, we repeated simulations using the complete 13-generation pedigree.

**Table 1:** Pair-wise relationships between individuals from our study of mammographic density (n = 1,481) and the HAPI Heart study (n = 868) after pedigree trimming.

	Number of Pairs	
Relationship Pair	Mammographic density study	HAPI Heart study
Parent-offspring	276	314
Siblings	1,254	592
Grandparent-grandchild	0	21
Avuncular	1,125	732
1st cousins	4,676	1,379
1st cousins, once removed	2,993	1,508
2nd cousins	1,345	905
Other	871	807

Note – Pedigree trimming yielded 177 families with 1–44 study participants per family (average of 8) in our study of mammographic density and 138 families with 1–46 study participants per family (average of 6) in the HAPI Heart study.

### Application of the Test to Empirical Data

We applied the RVKT to dozens of quantitative traits from the two genetic studies described above, with the goal of prioritizing traits and individuals for extreme-trait sequencing. Specifically, we applied the RVKT to 35 quantitative traits from our study of mammographic density (n = 1,481), including absolute measures of the dense and non-dense area of the breast, percent breast density, total breast size, measures of body size, reproductive and menstrual traits, and several serum hormones and growth factors (see Table S1). These traits, all of which are heritable, are of interest because of their associations with breast cancer risk. Prior to testing, we transformed each trait to approximate univariate normality, when necessary, and adjusted for age and menopausal status. For the hormones and growth factors, we carried out menopausal-specific analyses using batch-specific z-scores adjusted for age.

We also applied the RVKT to 37 quantitative traits from the HAPI Heart study (n = 868), including measures of body size, fasting lipid levels, and measures of vascular health and arterial stiffness (see Table S2). These traits, which are also heritable, are of interest because of their associations with cardiovascular disease. Prior to testing, we transformed each trait to approximate univariate normality, when necessary, and adjusted for age and sex.

As in our simulations, we tested tail sizes of 1, 2, 4, 6, and 8%, corresponding to 15, 30, 59, 89, and 118 subjects from our study of mammographic density and 9, 17, 34, 51, and 68 subjects from the HAPI Heart study. We then selected the RVKT statistic with the minimum p-value (p_min_) in the upper and lower tail of each trait distribution. To control for multiple testing of traits, some of which may be correlated, we calculated the effective number of tests using the method described by Li and Ji [18] and applied a Bonferroni correction to p_min_, denoted p_min, corrected_.

## Results

### Size of the Rare Variant Kinship Test

**Figure 1:**
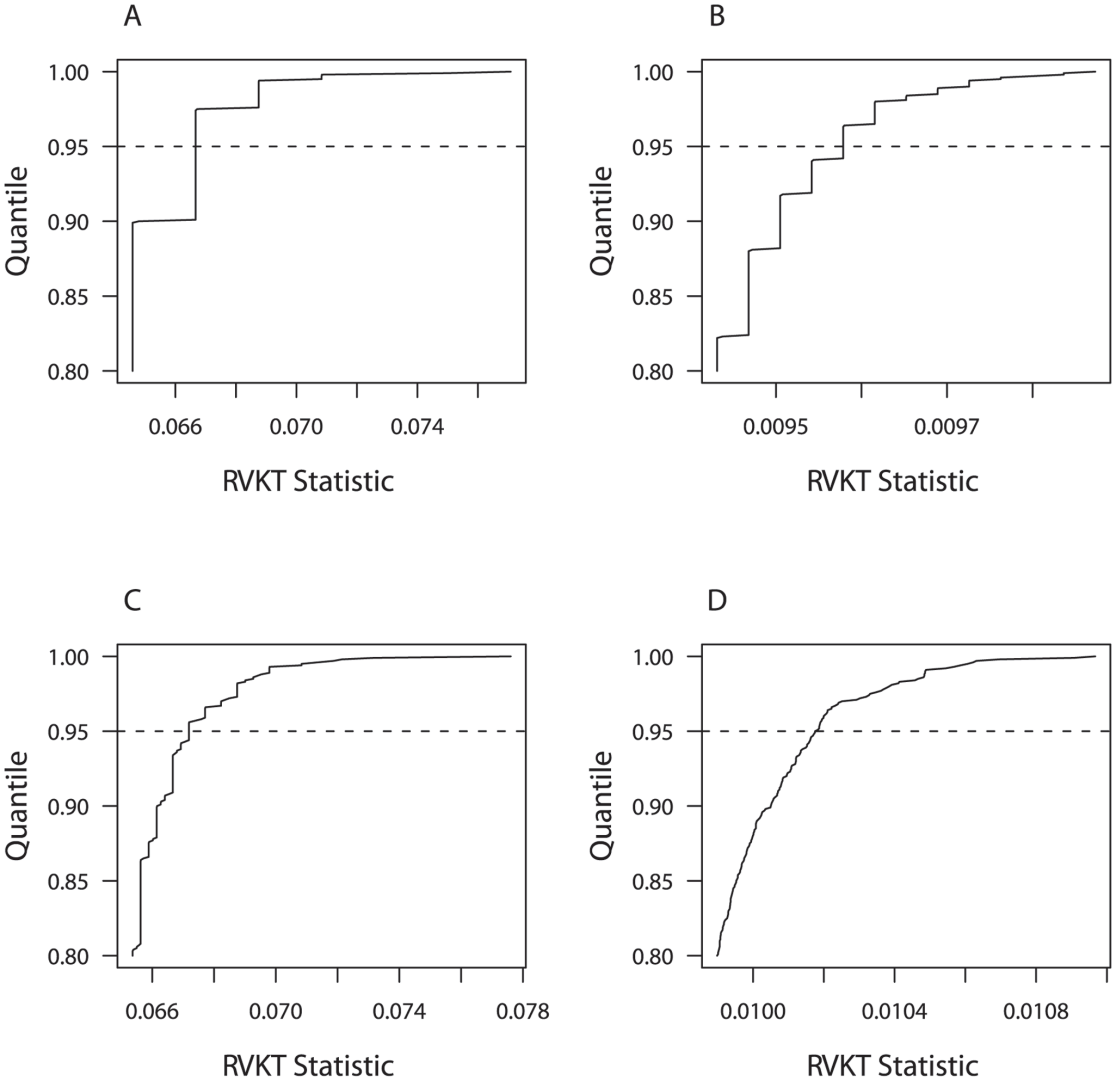
Top quintile of the cumulative distribution function of the RVKT statistic. Distribution is based on 1,000 replicates of a purely polygenic trait with a narrow-sense heritability of 40% and (panels A and B) four-person nuclear families (n = 1,484) or (panels C and D) trimmed pedigrees from our study of mammographic density (n = 1,481). Panels A and C are based on a tail size of 1% (15 individuals), and panels B and D are based on a tail size of 8% (118 individuals). Dashed line denotes the 95^th^ percentile.

To assess power, we used simulation to generate the null distribution of the RVKT statistic and determine the size (false-positive rate) of the test. As expected, the cumulative distribution function (CDF) was discrete. However, it became increasingly discrete as the number and types of relative pairs in the tail or sample decreased. For example, Figure 1 shows the top quintile of the CDF for a purely polygenic trait with a narrow-sense heritability of 40% and two sample structures: four-person nuclear families (n = 1,484) and trimmed Amish pedigrees from our study of mammographic density (n = 1,481). In the top quintile, the RVKT statistic assumed 36 values for the trimmed Amish pedigrees (Figure 1C) but only 6 values for nuclear families (Figure 1A), assuming a tail size of 1%. None of these values, however, coincided with the 95^th^ percentile of the CDF. Thus, in our power calculations below, we selected a rejection region having size as close as possible to 0.05, without exceeding 0.05, in order to maintain a significance level of 0.05.

### Power of the Rare Variant Kinship Test

Under the alternative hypothesis, power was generally maximized when the tail size matched the expected carrier frequency of the rare variant (data not shown). In other words, if *q* denotes the frequency of the rare variant, power was greatest for a tail size of 2*(1−q)q*+*q* ^2^. Thus, we report results below for tail sizes that maximized power.

**Figure 2:**
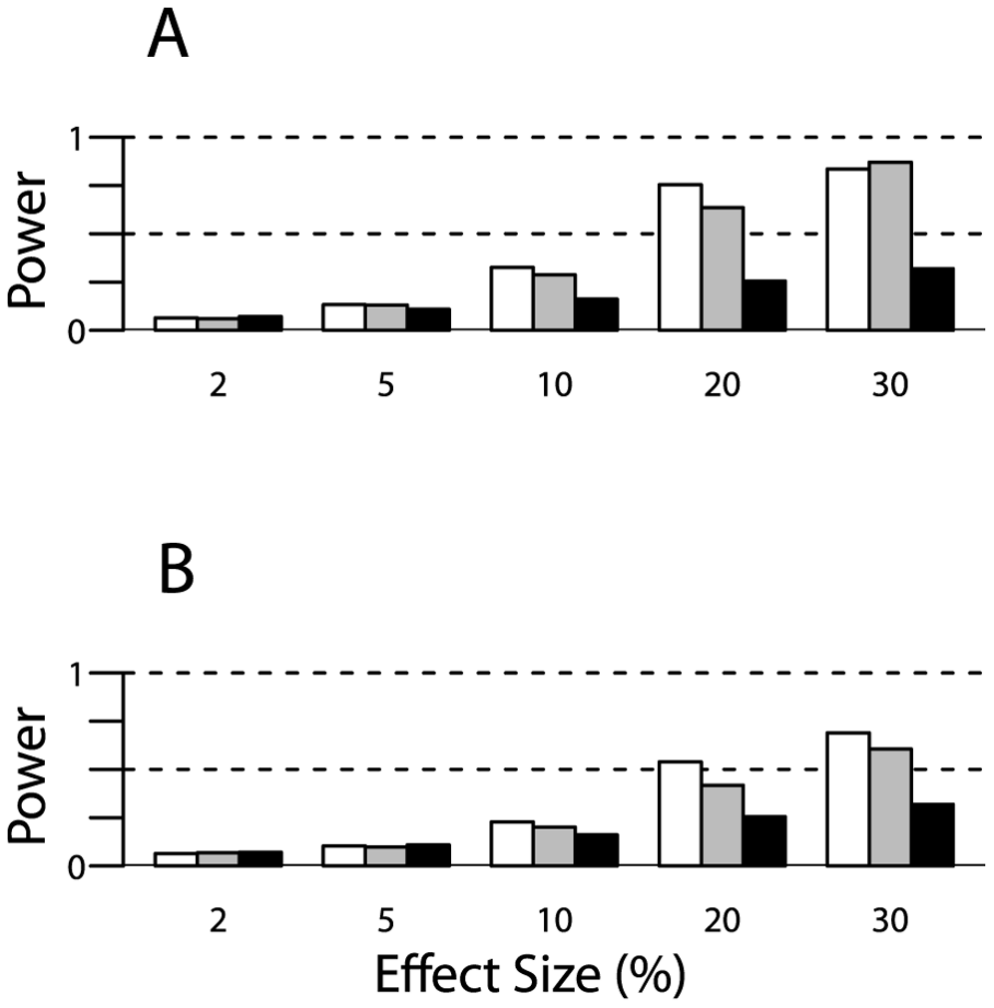
Power of the RVKT as a function of effect size. Effect size is the proportion of the trait variance explained by the rare variant. Results are based on 1,000 simulations of a quantitative trait and assume a rare variant allele frequency of 2%, a narrow-sense heritability of 40%, and pedigrees from (panel A) our study of mammographic density (n = 1,481) or (panel B) the HAPI Heart study (n = 868). Power is shown for trimmed Amish pedigrees (gray bars) and the complete 13-generation Amish pedigree (black bars). For comparison, power is also shown for four-person nuclear families (two parents and two offspring), with sample sizes equivalent to the sizes of our Amish studies (white bars). The significance level was set at 0.05.

**Figure 3:**
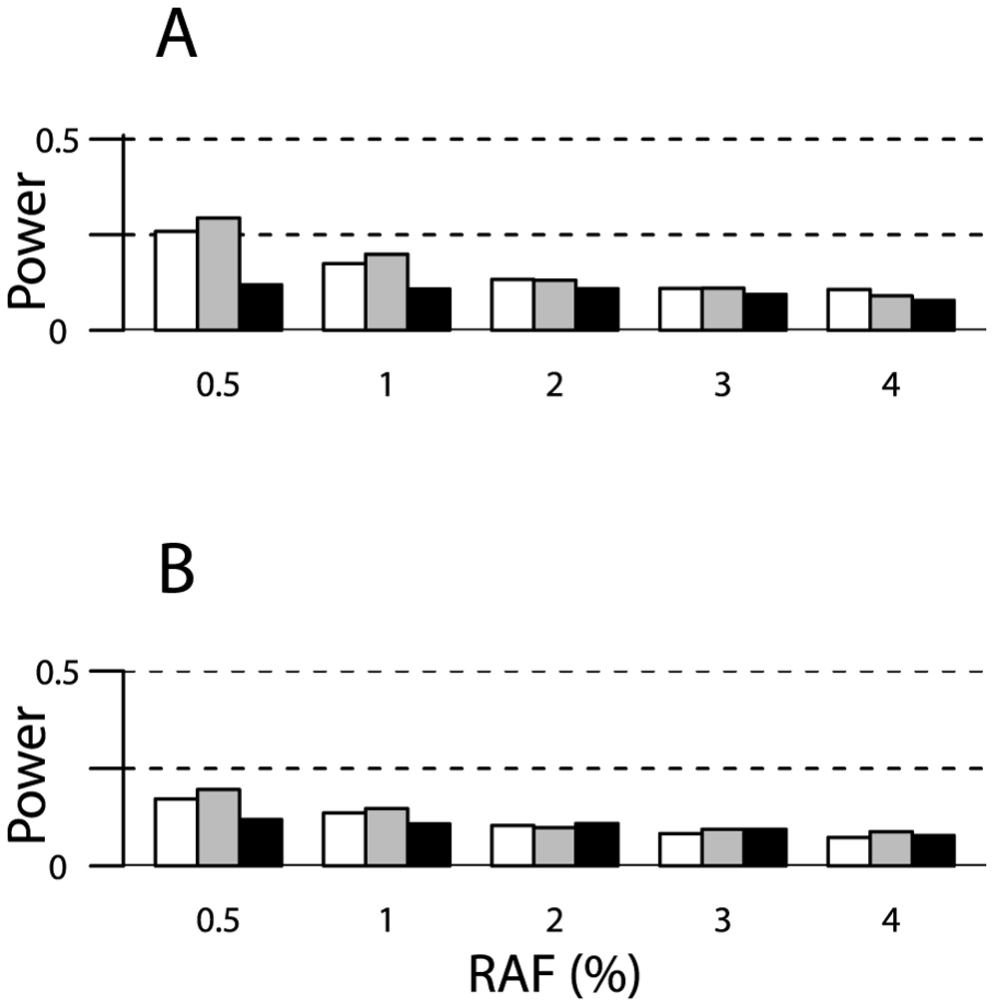
Power of the RVKT as a function of the rare variant allele frequency (RAF). Results are based on 1,000 simulations of a quantitative trait and assume a rare variant that accounts for 5% of the trait variance, a narrow-sense heritability of 40%, and pedigrees from (panel A) our study of mammographic density (n = 1,481) or (panel B) the HAPI Heart study (n = 868). Power is shown for trimmed Amish pedigrees (gray bars) and the complete 13-generation Amish pedigree (black bars). For comparison, power is also shown for four-person nuclear families (two parents and two offspring), with sample sizes equivalent to the sizes of our Amish studies (white bars). The significance level was set at 0.05.

**Figure 4:**
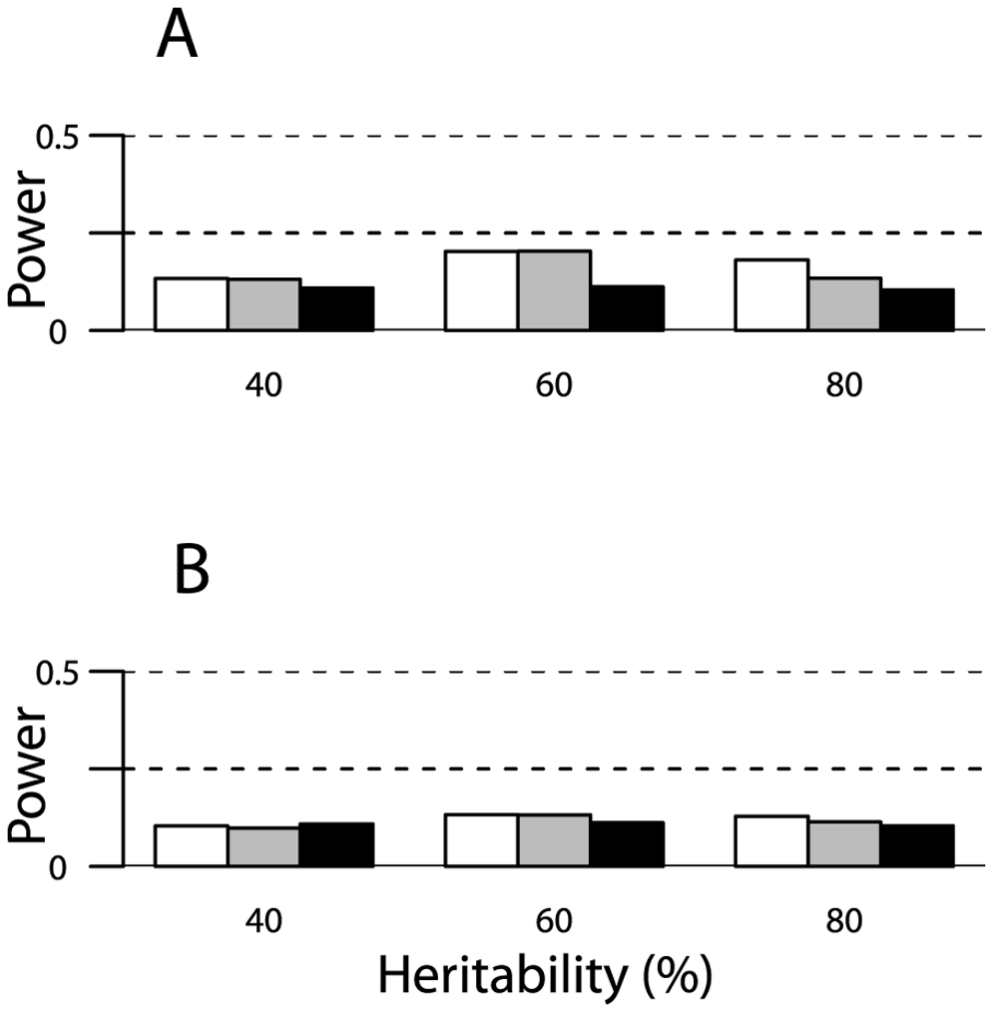
Power of the RVKT as a function of the narrow-sense heritability. Results are based on 1,000 simulations of a quantitative trait and assume a rare variant with an allele frequency of 2% that accounts for 1/8^th^ of the genetic variance and pedigrees from (panel A) our study of mammographic density (n = 1,481) or (panel B) the HAPI Heart study (n = 868). Power is shown for trimmed Amish pedigrees (gray bars) and the complete 13-generation Amish pedigree (black bars). For comparison, power is also shown for four-person nuclear families (two parents and two offspring), with sample sizes equivalent to the sizes of our Amish studies (white bars). The significance level was set at 0.05.

As expected, power of the RVKT increased as the effect size increased, meaning as the rare variant accounted for an increasing proportion of the trait variance. For example, using the trimmed pedigrees from our study of mammographic density (Table 1) and assuming a narrow-sense heritability of 40% and a rare variant frequency of 2%, power ranged from approximately 6 to 87% for effect sizes of 2 to 30%, respectively (Figure 2A). Similarly, based on the trimmed pedigrees from the HAPI Heart study and the same set of parameters, power ranged from approximately 7 to 61% (Figure 2B). As expected, power also increased as the sample size increased (Figure 2) and/or the rare variant frequency decreased (Figure 3). Power did not change much as the narrow-sense heritability of the trait varied from 40 to 80% (Figure 4).

Power degraded substantially as pedigree complexity increased, meaning as the number and types of distantly related pairs in a sample increased. For example, consider a sample of 1,481 individuals, a narrow-sense heritability of 40%, and a rare variant with frequency 2% and effect size 20%. Under these parameters, power decreased from 64% for the trimmed Amish pedigrees to 26% for the complete 13-generation pedigree (grey versus black bars in Figure 2A). In fact, power was actually higher with four-person nuclear families (n = 1,484 individuals; white bars in Figure 2A) than with our trimmed Amish pedigrees (75% versus 64%). Pedigree complexity also reduced power for smaller effect sizes (Figure 2) and for pedigree structures in the HAPI Heart study (Figure 2B).

### Application of the Rare Variant Kinship Test

**Figure 5:**
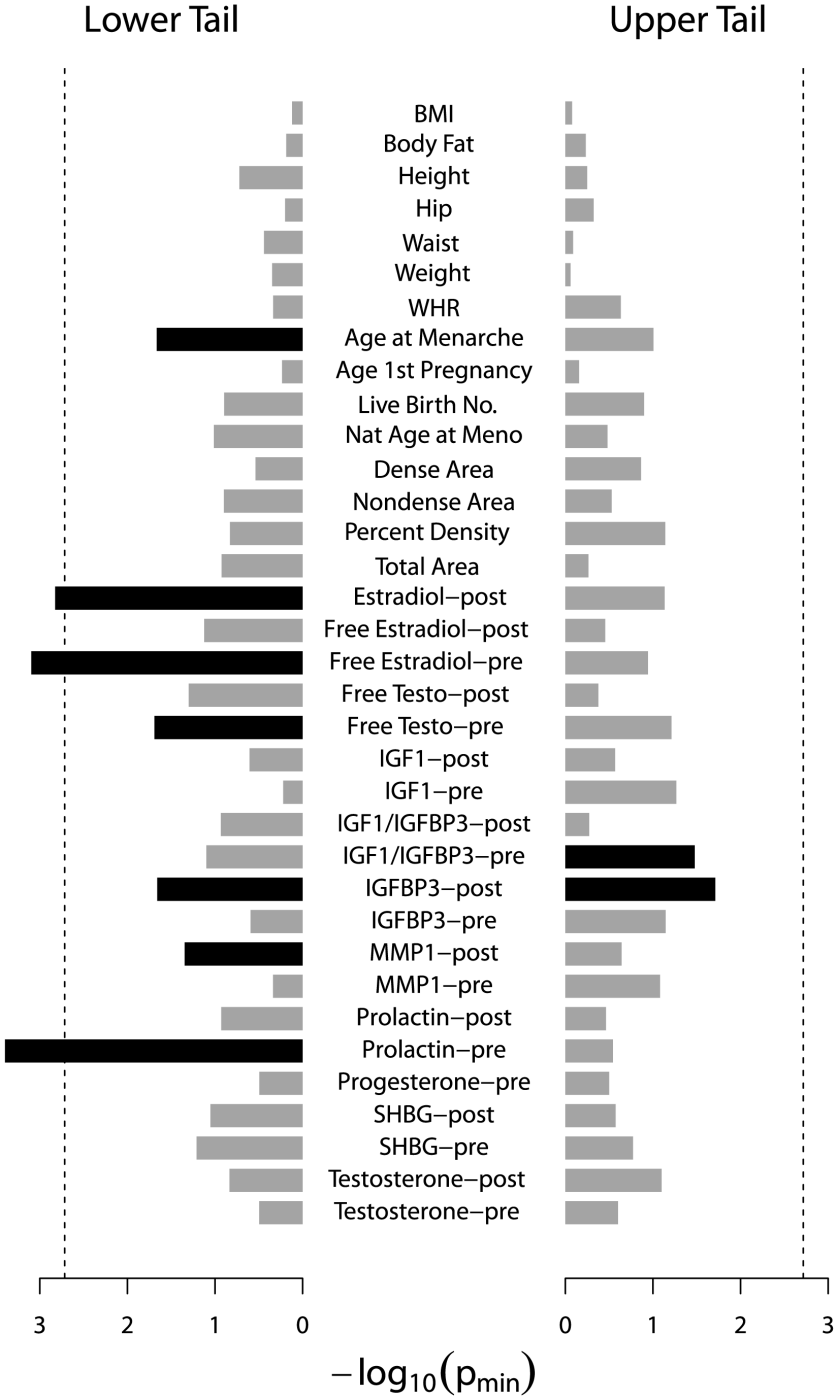
RVKT p-values (p_min_) for 35 quantitative traits from our study of mammographic density. Each bar represents the result for a single trait. Black bars, significant (p_min_≤0.05); gray bars, not significant. Dashed line denotes p-value threshold corrected for multiple testing. Before applying the RVKT, traits were transformed to approximate normality, when necessary, and adjusted for age and menopausal status, except for the hormones and growth factors, which were standardized by batch, adjusted for age, and analyzed separately for pre- and post-menopausal women.

**Figure 6:**
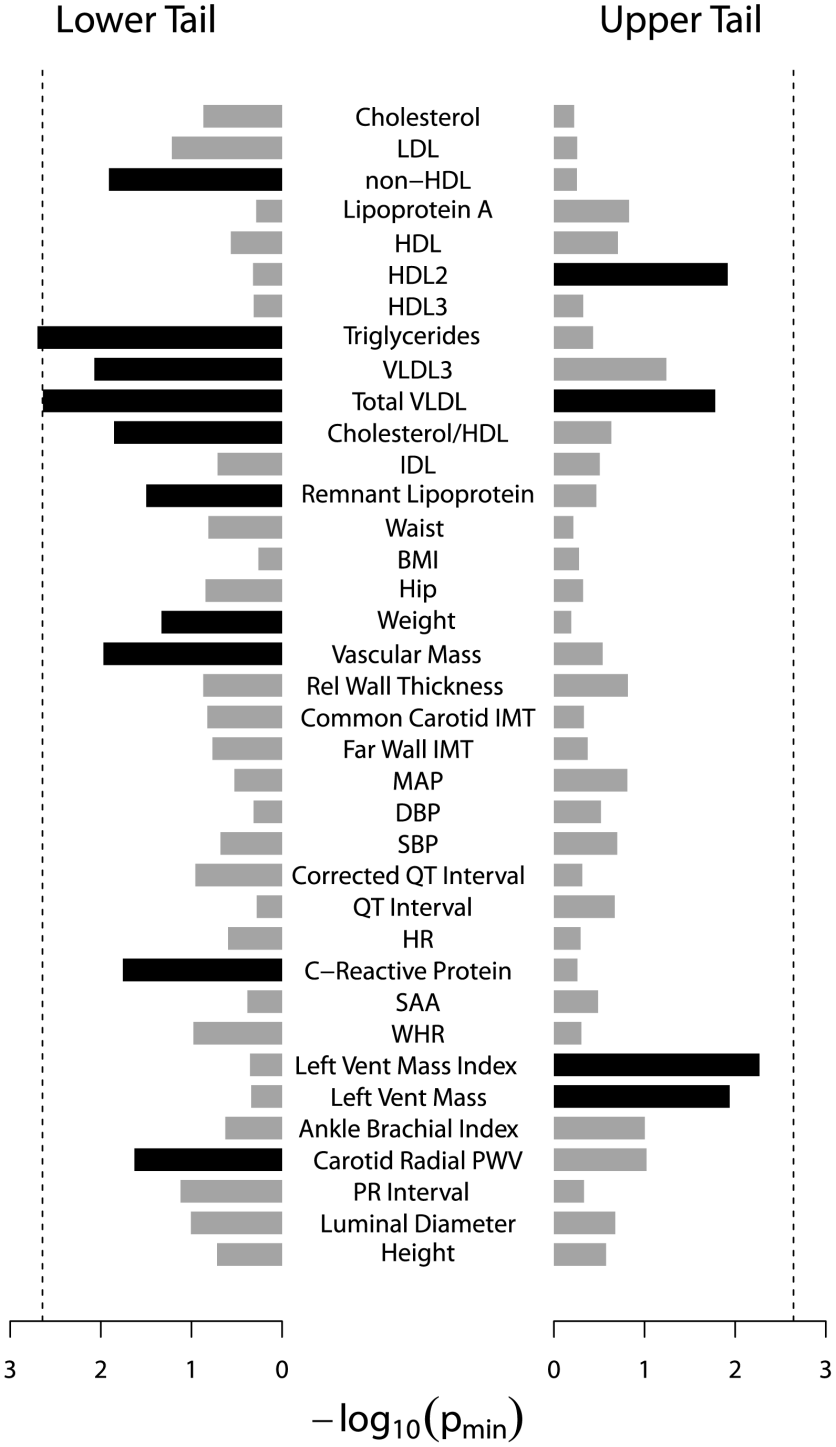
RVKT p-values (p_min_) for 37 quantitative traits from the HAPI Heart study. Each bar represents the result for a single trait. Black bars, significant (p_min_≤0.05); gray bars, not significant. Dashed line denotes p-value threshold corrected for multiple testing. Traits were transformed to approximate normality, when necessary, and adjusted for age and sex. Traits are ordered such that highly correlated traits are closer together.

After evaluating the power of the RVKT via simulation, we applied our test to dozens of quantitative traits from our two Amish studies. Figures 5 and 6 summarize RVKT p-values (p_min_) from our study of mammographic density and the HAPI Heart study, respectively. The RVKT statistic was nominally significant for 8 of the 35 traits in the density study (p_min_≤0.05). After correcting for multiple testing (26 effective tests), the RVKT remained significant for 3 of the 8 traits, including free estradiol and prolactin in pre-menopausal women and estradiol in post-menopausal women (p_min, corrected_≤0.05). Similarly, in the HAPI Heart study, the RVKT was nominally significant for 14 of the 37 traits, one of which, namely, fasting triglyceride levels, remained significant after correcting for 22 effective tests (p_min, corrected_ = 0.044).

In total, after multiple test correction, the RVKT statistic was significant for 4 of 72 quantitative traits across our two genetic studies. Table 2 gives results for each of these 4 traits for the tail size corresponding to the smallest empirical p-value (p_min_). For example, in pre-menopausal women from our study of mammographic density, p_min_, which corresponded to a tail size of 2% (14 of 728 women), was 0.0004 for prolactin. These 14 women had the lowest batch-standardized and age-adjusted levels of prolactin and a mean pair-wise kinship coefficient of 0.080 compared to an expected value of 0.068 under a purely polygenic model (approximate 95% confidence interval of 0.067 to 0.070). For each of the other 3 traits, the RVKT statistic was also significant when testing the lower but not upper tail of the trait distribution.

**Table 2:** Rare variant kinship test (RVKT) results from two genetic studies in the Amish.

Trait	Tail size (n)	Observed mean pair-wise kinship coefficient		Expected mean pair-wise kinship coefficient (approximate 95% CI)^d^	P-value^d^	
		Lower tail	Upper tail		Lower tail	Upper tail
Prolactin^a^	2% (14)	0.080	0.067	0.068 (0.067–0.070)	0.0004	1.0000
Free estradiol^a^	8% (57)	0.021	0.019	0.018 (0.017–0.019)	0.0008	0.1650
Estradiol^b^	6% (44)	0.026	0.025	0.024 (0.022–0.025)	0.0015	0.0737
Fasting triglycerides^c^	2% (17)	0.074	0.056	0.058 (0.056–0.063)	0.0020	0.7999

a Based on 728 pre-menopausal women from our study of mammographic density, and after standardizing by batch and adjusting for age, an estimated narrow-sense heritability of approximately 24% (for prolactin) and 34% (for free estradiol).

b Based on 753 post-menopausal women from our study of mammographic density, and after standardizing by batch and adjusting for age, an estimated narrow-sense heritability of approximately 35%.

c Based on 868 men and women from the HAPI Heart study, and after adjusting for age and sex, an estimated narrow-sense heritability of approximately 49%.

d Based on 10,000 simulations under the null hypothesis of a purely polygenic trait architecture.

## Discussion

The advantage of using the RVKT to prioritize traits and individuals for sequencing in family-based studies is best illustrated by results from the HAPI Heart study. In testing 37 quantitative traits, many of which are established risk factors for cardiovascular disease, we found significant evidence of excess relatedness between individuals in the lower tail of the distribution for fasting triglycerides. For tail sizes of 1 to 8%, the mean pair-wise kinship coefficient ranged from 0.114 to 0.020, respectively, and was significantly different from the kinship coefficient expected under a purely polygenic model of trait architecture (p≤0.05). Although differences between significance levels were not pronounced for different tail sizes, the significance of the RVKT was minimized for the 17 individuals with the lowest age- and sex-adjusted triglyceride levels, or equivalently, for a tail size of approximately 2%.

Remarkably, Pollin *et al.* [19] previously identified a null mutation in the *APOC3* gene (R19X) (rs76353203) with a frequency of 0.024 that lowers fasting triglyceride levels in HAPI Heart study participants. This mutation was discovered because it was tagged by another SNP (rs10892151) (MAF = 0.028) in the context of a GWAS (p = 4.1×10^−13^, r^2^ = 0.85 between rs76353203 and rs10892151). Had we sequenced the 17 individuals in the lower tail of the age- and sex-adjusted triglyceride distribution, we would have discovered *APOC3* R19X since 7 of these individuals were mutation carriers, an 8-fold enrichment compared to the ∼5% of individuals who were carriers in the overall sample. Notably, none of the 17 individuals in the upper tail of the distribution carried the mutation.

As expected, the power of the RVKT was low for small to modest effect sizes. In fact, the power of our test to implicate the presence and influence of *APOC3* R19X on fasting triglycerides in the HAPI Heart study was less than 25%. As such, it cannot be used to exclude the presence of rare trait-associated alleles, unless these alleles account for a large proportion of the phenotypic variance. However, when multiple medically relevant quantitative traits are available, the RVKT may be a valuable starting point for prioritizing traits and individuals for sequencing. For example, even though *APOC3* R19X carriers in the HAPI Heart study had cardio-protective profiles for several lipids, including higher HDL and lower LDL cholesterol and lower triglyceride levels, Pollin et al. [19] discovered R19X because its tag SNP had an exclusive genome-wide significant association with fasting triglyceride levels. Consistent with their findings, we singled out fasting triglycerides – out of 37 traits – as the basis for an extreme-trait sequencing study by applying the RVKT.

The power of the RVKT is heavily influenced by the number and types of relationships in a sample. Specifically, the power of the RVKT increases as the number of closely related pairs increases. In contrast, power is lost as the number of distantly related pairs multiplies. For instance, in our simulations (Figures 2–4), power was actually greater with the trimmed Amish pedigrees than with the complete 13-generation pedigree, with differences as great as 20–30% for large effect sizes. To understand why, it’s helpful to consider the impact of trimming on the mean kinship coefficient under the null and alternative hypotheses. Under both hypotheses, trimming decreases the mean since individuals who are distantly related, say third cousins, appear to be unrelated. However, it does so to a lesser extent under the alternative hypothesis. This is because the mean under the alternative is dominated by closely related pairs, which are maintained regardless of trimming. As a result, the difference between the mean kinship coefficient under the null and alternative hypotheses is larger – and in turn, power is greater – with trimming than without.

Although trimming increases power, the RVKT may actually be conservative when pedigrees are too simple. In fact, it may be impossible to choose a rejection region from the empirical null distribution of the RVKT statistic such that the size of the test does not exceed the significance level. For example, consider a single pair of siblings from each of 741 families (n = 1,482). To obtain the null distribution, we simulated 1,000 replicates of a purely polygenic trait with a narrow-sense heritability of 40%. However, when we tested a 1% tail size, we obtained only three values of the RVKT statistic (data not shown). The largest value occurred 17 times; therefore, the smallest possible test size was 0.017. In other words, it would have been impossible to conduct a 0.01 level test. This problem was especially pronounced for modest sample sizes and small tail sizes due to discontinuities in the empirical null distribution of the mean pair-wise kinship coefficient (data not shown).

An implicit assumption of the RVKT is that – within each family – a specific allele at the same locus has an effect on the trait of interest. In other words, the power of the test depends on the extent of allelic and locus homogeneity within each family but does not require homogeneity between families. For example, if multiple rare variants influence a trait, then phenotypically extreme individuals from the same family are more likely to share the same trait-influencing alleles than phenotypically extreme individuals from different families. Thus, the RVKT statistic may still exceed its expected value since individuals in the tail of the trait distribution may be more closely related than expected under the null hypothesis. From this perspective, isolates like the Amish are an ideal population in which to apply the RVKT and carry out extreme-trait sequencing since many copies of the same rare trait-associated allele are likely to be segregating within a family due to a combination of founder effect and genetic drift.

In our simulations, we considered rare variant frequencies ranging from 0.5 to 4%. We did so for two reasons. First, the Old Order Amish population of Lancaster County, PA derives from a small number of European ancestors (∼300) who immigrated nearly 250 years ago and has since increased in size to approximately 45,000 individuals (census size) [20]. Thus, many alleles that were initially rare or private in the ancestral population, e.g., MAF<0.5% in HapMap or 1KG projects, have either been eliminated from the Amish or increased in frequency due to founder effect and/or genetic drift. Second, even in the presence of the allelic heterogeneity typical of non-founder populations, the aggregate trait-associated allele frequency at a single locus may still be greater than 0.5% and thus potentially amenable to detection by the RVKT.

Prioritizing traits and individuals for sequencing using the RVKT requires only pedigree and phenotype data and thus can be done before carrying out costly sequencing experiments. This process, however, requires accurate pedigree and phenotype data. Likewise, it is important to consider the impact of adjusting for covariates or stratifying the analysis by subgroups before identifying individuals with extreme trait values. For example, in our study of mammographic density, we found significant evidence for the presence of rare variants influencing the dense area of the breast in post- but not pre-menopausal women. Specifically, after adjustment for age, p_min_ for the RVKT was 0.018 and 0.031 for the lower and upper tails, respectively (see Figure S1). These results suggest the presence of at least one variant that lowers density and another variant that increases density in post-menopausal women.

We developed the RVKT to inform the selection of traits and individuals for sequencing and rare variant discovery. Predictably, the power of our test depended – above all – on the effect size of the rare variant. Indeed, it was underpowered to detect rare variants unless those variants had large effects. However, our analysis of over 70 quantitative traits from our Amish studies suggests that the results may still be informative to prioritize sequencing efforts.

## Supporting Information

Figure S1 **RVKT p-values (p_min_) for quantitative traits from our study of mammographic density stratified by menopausal status.** Each bar represents the result for a single trait. Black bars, significant (p_min_≤0.05); gray bars, not significant. Before applying the RVKT, traits were transformed to approximate normality, when necessary, and adjusted for age.
(TIF)

Table S1 **Trait descriptions from our study of mammographic density.**
(PDF)

Table S2 **Trait descriptions from the HAPI Heart study.**
(PDF)
