# Supplementary figures and images for: A Method to Prioritize Quantitative Traits and Individuals for Sequencing in Family-Based Studies
Source: PLoS One. 2013 Apr 23;8(4):e62545. doi: 10.1371/journal.pone.0062545 (PMC3633859; doi:10.1371/journal.pone.0062545)

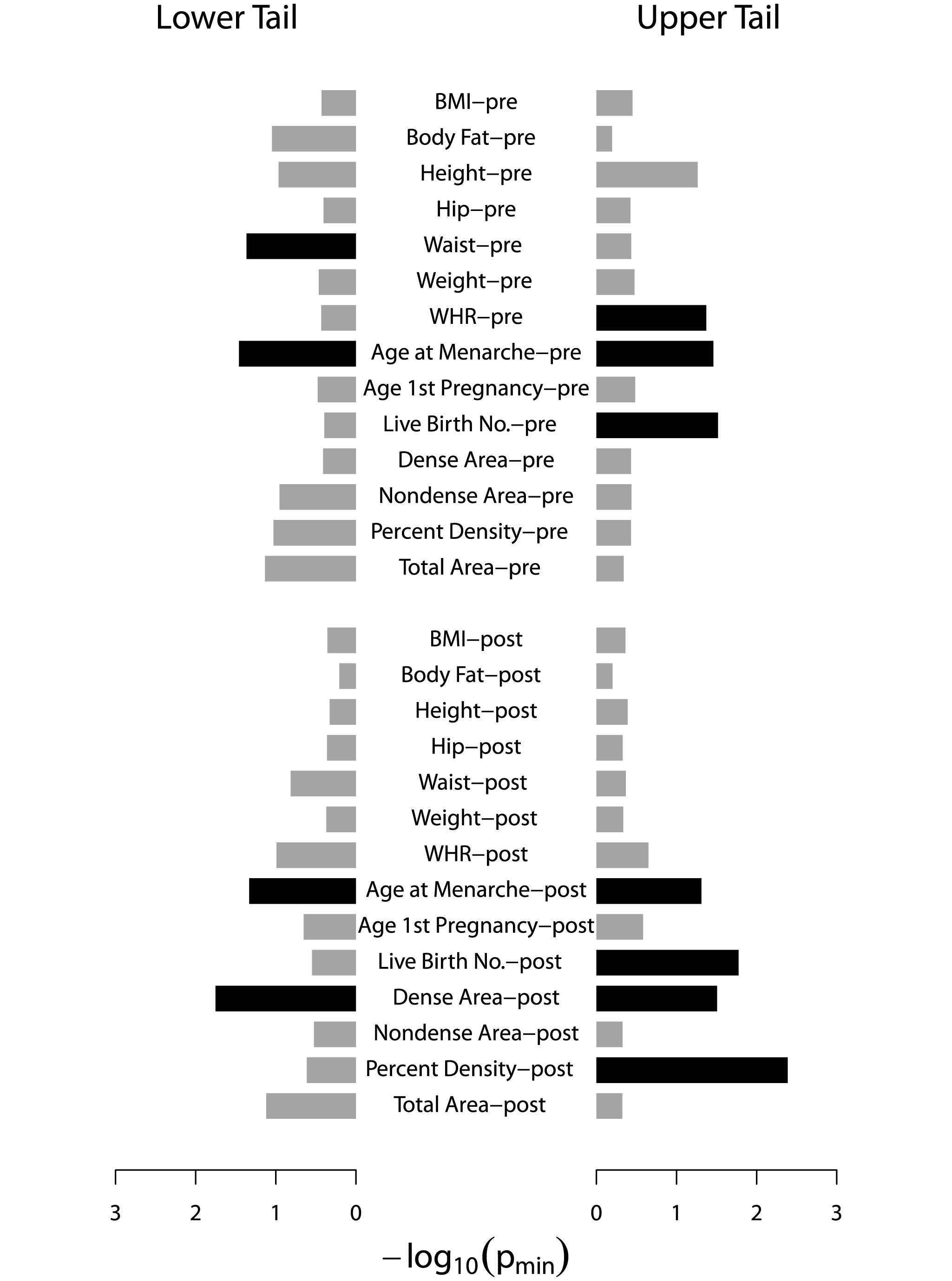

Supplement: Figure S1 — RVKT p-values (pmin) for quantitative traits from our study of mammographic density stratified by menopausal status. Each bar represents the result for a single trait. Black bars, significant (pmin≤0.05); gray bars, not significant. Before applying the RVKT, traits were transformed to approximate normality, when necessary, and adjusted for age. (TIF) [file pone.0062545.s001.tif]
