# Supplementary material for: A Method to Prioritize Quantitative Traits and Individuals for Sequencing in Family-Based Studies
Source: PLoS One. 2013 Apr 23;8(4):e62545. doi: 10.1371/journal.pone.0062545 (PMC3633859; doi:10.1371/journal.pone.0062545)
Supplement: Table S1 — Trait descriptions from our study of mammographic density. (PDF) [file pone.0062545.s002.pdf]

Table S1. Trait descriptions from our study of mammographic density

Trait	Description	N	h^2
BMI	Body Mass Index (kg/m^2)	1,481	0.48
Body Fat	Percent body fat	1,109	0.52
Height	Height (cm)	1,481	0.94
Hip	Hip circumference (cm)	1,481	0.21
Waist	Waist circumference (cm)	1,481	0.52
Weight	Weight (kg)	1,481	0.57
WHR	waist circumference/ hip circumference	1,481	0.54
Age at Menarche	Age at first menstrual period	1,479	0.54
Age 1st Pregnancy	Age at first child	1,349	0.25
Live Birth No.	Number of live births	1,355	0.40
Nat Age at Meno	Age at natural menopause	671	0.42
Dense Area	Dense breast area (cm^2)	1,481	0.49
Nondense Area	Non-dense breast area (cm^2)	1,481	0.70
Percent Density	Percent density = dense area/total area * 100	1,481	0.45
Total Area	Total breast area (cm^2)	1,481	0.75
Estradiol-post	Estradiol (pg/mL) in postmenopausal women	752	0.35
Free Estradiol-post	Estradiol indexed for SHBG in postmenopausal women	752	0.28
Free Estradiol-pre	Estradiol indexed for SHBG in premenopausal women	728	0.34
Free Testo-post	Testosterone indexed for SHBG in postmenopausal women	752	0.50
Free Testo-pre	Testosterone indexed for SHBG in premenopausal women	728	0.72
IGF1-post	Insulin-like growth factor 1 (ng/mL) in postmenopausal women	546	0.77
IGF1-pre	Insulin-like growth factor 1 (ng/mL) in premenopausal women	477	0.60
IGF1/IGFBP3-post	IGF1/IGFBP3 in postmenopausal women	546	0.74
IGF1/IGFBP3-pre	IGF1/IGFBP3 in premenopausal women	477	0.31
IGFBP3-post	Insulin-like growth factor binding protein 3 (ng/dL) in postmenopausal women	547	0.60
IGFBP3-pre	Insulin-like growth factor binding protein 3 (ng/dL) in premenopausal women	477	0.69
MMP1-post	MMP-1 measured in serum in postmenopausal women	693	0.55
MMP1-pre	MMP-1 measured in serum in premenopausal women	668	0.87
Prolactin-post	Prolactin (ng/mL) in postmenopausal women	752	0.26
Prolactin-pre	Prolactin (ng/mL) in premenopausal women	728	0.24

Progesterone-pre	Progesterone (ng/mL) in premenopausal women	728	0.35
SHBG-post	Steroid hormone binding globulin (nM) in postmenopausal women	752	0.33
SHBG-pre	Steroid hormone binding globulin (nM) in premenopausal women	728	0.70
Testosterone-post	Testosterone (ng/dL) in postmenopausal women	752	0.50
Testosterone-pre	Testosterone (ng/dL) in premenopausal women	728	0.55

Note – h^2 is the narrow sense heritability of each trait after adjusting for age and menopausal status, and for the hormones and growth factors, after standardizing by batch, adjusting for age, and stratifying analyses by menopausal status; h^2 was significantly different from 0 ($p \leq 0.05$) for all traits except estradiol in premenopausal women and progesterone in postmenopausal women
