# Supplementary material for: A Method to Prioritize Quantitative Traits and Individuals for Sequencing in Family-Based Studies
Source: PLoS One. 2013 Apr 23;8(4):e62545. doi: 10.1371/journal.pone.0062545 (PMC3633859; doi:10.1371/journal.pone.0062545)
Supplement: Table S2 — Trait descriptions from the HAPI Heart study. (PDF) [file pone.0062545.s003.pdf]

Table S2. Trait descriptions from the HAPI Heart study

Trait	Description	N	h^2
BMI	Body Mass Index (kg/m^2)	868	0.49
Height	Height (cm)	868	0.71
Hip	Hip circumference (cm)	868	0.42
Waist	Waist circumference (cm)	868	0.51
Weight	Weight (kg)	868	0.59
WHR	Waist circumference/ hip circumference	868	0.38
Cholesterol	Fasting Total Cholesterol (mg/dl)	858	0.73
Cholesterol/HDL	Total cholesterol/HDL cholesterol	858	0.59
C-Reactive Protein	C-Reactive Protein levels	857	0.33
HDL	Fasting HDL Cholesterol (mg/dl)	858	0.58
HDL2	Fasting HDL sub fraction 2 (mg/dl)	850	0.51
HDL3	Fasting HDL sub fraction 3 (mg/dl)	850	0.50
IDL	Fasting intermediate density lipoprotein (mg/dl)	850	0.44
LDL	Fasting LDL Cholesterol (mg/dl)	857	0.73
Lipoprotein A	Fasting lipoprotein A	850	0.62
non-HDL	Fasting non-HDL cholesterol (mg/dl)	850	0.68
Remnant Lipoprotein	Fasting remnant lipoprotein cholesterol	849	0.46
SAA	Serum Amyloid A	510	0.34
Total VLDL	Fasting very low density lipoprotein cholesterol (mg/dl)	850	0.42
Triglycerides	Fasting triglycerides (mg/dl)	858	0.50
VLDL3	Fasting VLDL sub fraction 3 (mg/dl)	850	0.44
Corrected QT Interval	QT Interval from the EKG corrected for heart rate	866	0.52
DBP	Diastolic blood pressure (mmHg)	868	0.14
HR	Heart rate	866	0.19
MAP	Mean arterial pressure = 2/3 DBP + 1/3 SBP	868	0.21
PR Interval	PR Interval from an EKG	799	0.38
QT Interval	QT Interval from the EKG	866	0.26
SBP	Systolic blood pressure (mmHg)	868	0.32
Carotid Radial PWV	Pulse wave velocity in the radial carotid	664	0.18
Common Carotid IMT	Common carotid artery Intimal Medial Thickness	819	0.33
Far Wall IMT	Common carotid artery Intimal Far Wall Max Thickness, mean of 4 measures (mm)	809	0.37
Left Vent Mass	Left ventricle mass measured at echocardiogram	835	0.28
Left Vent Mass Index	Left Ventricular Mass / Body Surface Area	835	0.24
Luminal Diameter	Diameter of the common carotid artery at the end	809	0.53

	diastole (mm)		
Ankle Brachial Index	Average of right and left ankle brachial index (mmHg)	861	0.23
Rel Wall Thickness	CommonCarotidIMT / LuminalDiameter	809	0.24
Vascular Mass	1.06*pi*((LuminaDiameter/2 + CommonCarotidIMT)^2 - (LuminalDiameter/2)^2) (cm^2)	809	0.29

Note – h^2 is the narrow sense heritability of each trait after adjusting for age and sex; h^2 was significantly different from 0 ($p \leq 0.05$) for all traits
